# Supplementary material for: Ultrasensitive proteome analysis using paramagnetic bead technology
Source: Mol Syst Biol. 2014 Oct 30;10(10):1–10. doi: 10.15252/msb.20145625 (PMC4299378; doi:10.15252/msb.20145625)
Supplement: Supplementary file 2 — Supplementary Tables [file msb0010-0757-sd2.pdf]

Supplementary Information for

Ultrasensitive proteome analysis using paramagnetic bead technology

Christopher S Hughes, Sophia Foehr, David A Garfield, Eileen E Furlong, Lars M Steinmetz, Jeroen Krijgsveld*

European Molecular Biology Laboratory, Genome Biology Unit, Heidelberg, Germany

Table of contents

Supplementary methods……………………………………………………….....2
Cell culture……………………………………………………………………2
Cell Lysis, Protein Reduction, and Alkylation………………………….......3
FASP treatment…………………………………………………………….4
Optimization of SP3 for protein enrichment………………………….…….5
Optimization of SP3 for peptide enrichment…………………………..……8
Chemical Labeling of Peptides in SP3……………………………….…….9
Alternative Peptide Clean-up Procedures………………………………10
High-pH Reversed Phase Fractionation……………………………...11
SP3 fractionation………………………………………………………….11
Mass Spectrometry Analysis…………………………………………….12
Mass Spectrometry Data Analysis……………………………………13
Bioinformatic and Statistical Analyses…………………………………..14
Supplementary Protocols………………………………………………………17
Reagents & Equipment…………………………………………………17
Bead Preparation………………………………………………………17
Cell Lysis and Protein Preparation for *Drosophila* Embryos…………..18
SP3 Protein Clean-up……………………………………………………..19
SP3 Peptide Recovery and Preparation……………………………...20
Supplementary Figures S1-S13……………………………………………..22
Supplementary References...……………………………………………………36

Supplementary Methods

Cell Culture

The yeast strain YAL6B (MATa, his3Δ leu2Δ met15Δ lys1::KanMX6 arg4::KanMX4)(Gruhler *et al*, 2005) was cultured in rich medium (YPD) for all experiments. Replicate cultures were harvested at an optical density (OD600) of ~0.8. Cells were harvested through centrifugation, rinsed with ice-cold PBS and snap frozen until use.

HeLa Kyoto cells were cultured in DMEM with Glutamax supplemented with 10% fetal bovine serum and 1X non-essential amino acids. Cells were cultured at 37°C in a 5% CO_2 environment. Cells were harvested through incubation with a solution of 0.05% trypsin-EDTA, and centrifuged. Recovered cells were rinsed and counted prior to aliquoting to the desired cell number per tube. Approximate cell counts were acquired using a haemocytometer. Cell pellets were always directly lysed with no snap freezing. 46C mouse embryonic stem cells (mESC) were cultivated in medium containing Knockout DMEM supplemented with 15% Knockout serum replacement, 2mM GlutaMAX, 1X non-essential amino acids, 0.1 mM beta-mercaptoethanol, 8 μg/mL leukemia inhibitory factor, 100 U/mL penicillin, and 100 mg/mL streptomycin. mESCs were differentiated to neural progenitor (NP) cells through culture in N2B27 medium composed of DMEM/F12, 1x N2-supplement, and 0.25 mg/mL bovine serum albumin fraction V. This was combined 1:1 with Neurobasal medium supplemented with 1x B27-supplement, 1 mM GlutaMAX, 100 U/mL penicillin, and 100 mg/mL streptomycin. mESCs and NP cells were cultured on dishes coated

with 0.2% gelatin. All cell culture reagents were obtained from Life Technologies unless noted otherwise.

Cell Lysis, Protein Reduction, and Alkylation

Frozen yeast cell pellets were lysed using a mechanical disruption approach. Lysis buffer was composed of 1% SDS (Bio-Rad), 5mM dithiothreitol (DTT) (Bio-Rad), 1X cOmplete Protease Inhibitor Cocktail-EDTA (Roche), prepared in 50 mM HEPES buffer at pH 8.5 (Sigma). A pellet equivalent to 50 mL of ~0.8 OD600 cells was combined with 500 µL of lysis buffer in a 2 mL screw-cap tube containing a 500 µL equivalent of acid-washed glass beads (Sigma, 425 – 600 µm). Tubes were incubated for 5 minutes at 95°C and subsequently placed on ice for 5 minutes. Tubes were shaken vigorously on a Fast-Prep instrument (MP Biomedical) for 45 seconds at speed 5. Vials were then sonicated in a Bioruptor (Diagenode) for 15 cycles (30 seconds on, 30 seconds off) on the setting 'high' with chilling set at 4°C. Lysates were then recovered into 2mL tubes by piercing the bottom of the screw cap tube with a heated syringe and centrifuging for 1 minute at 3,000g. Cell debris was pelleted with further centrifugation at 15,000g for 10 minutes. Recovered lysates were stored at -80°C until use.

HeLa, mESC, and NP cells were lysed using a solution-based procedure without physical disruption. Specific numbers of harvested cells were diluted in phosphate buffered saline (PBS) such that the concentration did not exceed 25,000 cells in 5 µL. Lysis buffer was composed of 1% SDS (Bio-Rad), 1X cOmplete Protease Inhibitor Cocktail-EDTA (Roche), prepared in 50mM HEPES buffer at pH 8.5 (Sigma). Lysis was induced through addition of an equal volume of lysis buffer. Mixtures were heated for 5

minutes at 95°C and subsequently placed on ice for 5 minutes. To each tube, 25 Units of Benzonase Nuclease (Novagen, CAT# 70664) was added per 500,000 cells to degrade chromatin. Tubes were incubated at 37°C for 30 minutes in a thermocycler. This is a critical step in the lysis protocol, as excess intact-chromatin will disrupt SP3.

Proteins in the yeast, HeLa, mESC, and NP solutions were reduced through the addition of 5 µL of 200mM DTT (Bio-Rad) per 100 µL of lysate. *Drosophila* embryo lysates contained sufficient DTT for reduction in the lysis buffer. Tubes were incubated at 45°C for 30 minutes in a thermocycler to promote disulfide reduction. Alkylation was performed through the addition of 10 µL of 400mM iodoacetamide (IAA) per 100 µL of lysate. Tubes were incubated at 24°C in the dark for 30 minutes in a thermocycler. Reactions were quenched through the addition of 10 µL of 200 mM DTT per 100 µL of lysate.

FASP Treatment

FASP was carried out as described previously(Wisniewski *et al*, 2009) with minor modifications. Briefly, each reduced and alkylated protein mixture was diluted to a final volume of 30 µL with 50mM HEPES at pH 8. To this, 200 µL of 8M urea was added to the protein sample and centrifuged in Vivacon (Sartorius) 30 kDa molecular weight cut-off filters for 10 minutes at 14,000g. This step was repeated a further 3 times. Filters were then rinsed 3 times with 100 µL of 50 mM HEPES, pH 8 by centrifugation. Proteolysis was carried out overnight in-filter in a wet-chamber at 37°C with a mixture of trypsin and rLysC (Promega) at an enzyme to substrate ratio of 1:25. Peptides were eluted with sequential rinses of 50mM HEPES (2 times) and

200mM NaCl (1 time). Eluted peptides were desalted using SepPaks (Waters) prior to MS analysis.

Optimization of SP3 for protein enrichment

In all protein experiments, 2 μL of a 10 μg/μL stock of beads was used as discussed in the 'Protein SP3 Protocol' methods section in the main text. All reactions were performed in PCR tubes (Ratiolab).

To determine the optimum concentration of acetonitrile to promote protein binding to the beads, 5 μL (~10 μg of protein) aliquots of the yeast lysate prepared in 1% SDS containing buffer as described above were used. Equivalent bead-protein mixtures were either acidified using formic acid, or left at neutral pH. To the bead-protein mixtures, acetonitrile was added from a 100% stock to adjust the final concentration in steps of 5% between 20 – 95% for individual samples. Mixtures were incubated for 8 minutes at room temperature, and then placed on a magnetic rack for a further 2 minutes. Beads were rinsed twice with 200 μL of 70% ethanol for 30 seconds each and the supernatants discarded. Beads were then rinsed with 180 μL of 100% acetonitrile for 15 seconds and the supernatant discarded. Proteins were eluted into 5 μL of 50mM HEPES pH 8 that contained ~250 ng of Trypsin/rLysC enzyme mix (Promega). Digests were incubated for 14 hours at 37°C. Resultant bead-peptide mixtures were pipette mixed to resuspend settled beads. To each tube, acetonitrile from a 100% stock was added to a final concentration >95% and incubated for 8 minutes, followed by 2 minutes on a magnetic rack. Beads were rinsed once with 180 μL of acetonitrile, and peptides eluted off the beads in 10 μL of 2% DMSO with pipette mixing for 5 minutes. Samples were acidified with formic acid prior to MS-analysis. The

signal intensity and complexity between runs was compared to determine the optimum percentage of acetonitrile. A final concentration of 50% of acetonitrile in acidic conditions was found to give the highest and most reproducible recovery.

Using this optimized condition, SP3 was compared with conventional methods for protein clean-up and enrichment. To observe protein recovery, equivalent aliquots of the yeast cell lysate (~10 μg of protein) were loaded on the gel with or without (Control) prior treatment with SP3. Before loading on the gel, samples were diluted in Laemlli buffer. Gels were 12% Tris-glycine cast in-house. Gels were photographed and recovery compared using densitometry obtained with a Bio-Rad GS-800 scanner.

SP3 was also compared with alternative methods of protein enrichment using MS-analysis instead of SDS-PAGE: 1. Nanodiamond conditions, 2. SPRI precipitation conditions, and 3. Treatment with FASP protocol as discussed above. In nanodiamond conditions, bead-protein mixtures were acidified with 5 μL of 1% formic acid. In SPRI conditions, bead-protein mixtures were mixed with 15 μL of precipitation mix (2.5M NaCl, 20% ammonium sulfate). In both conditions, mixtures were incubated for 8 minutes at room temperature, and for a further 2 minutes on a magnetic stand. Beads were rinsed as before (2X 70% Ethanol, 1X 100% acetonitrile) and eluted in 5 μL of 50 mM HEPES pH 8 containing ~250 ng of Trypsin/LysC enzyme mix. Digests were incubated for 14 hours at 37°C. Resultant bead-peptide mixtures were pipette mixed to resuspend settled beads. To each tube, acetonitrile from a 100% stock was added to a final concentration >95% and incubated for 8 minutes, followed by 2 minutes on a

magnetic rack. Beads were rinsed once with 180 µL of acetonitrile, and peptides eluted off the beads in 10 µL of 2% DMSO with pipette mixing for 5 minutes. Samples were acidified with formic acid prior to MS-analysis.

To determine the approximate binding capacity of the beads bovine serum albumin (BSA) was used. A stock solution of 50 mg/mL was prepared in 1% SDS (Bio-Rad), 1X cOmplete Protease Inhibitor Cocktail-EDTA (Roche), in 50mM HEPES buffer at pH 8.5 (Sigma). The BSA was heated at 95°C for 5 minutes, placed on ice, reduced with DTT and alkylated with IAA as above. The capacity was tested using 1 µg of beads from a 10 µg/µL stock prepared with 1 µg, 100 µg, 200 µg, and 400 µg of reduced and alkylated BSA in 1% SDS containing buffer. The mixtures were treated with SP3 using 50% acetonitrile under acidic conditions as described above. The recovered protein was digested and resulting peptides diluted such that the concentration was equal based on the starting amount of BSA. Recovery was measured using MS intensity and peak matching between samples and a standard BSA peptide mixture of known concentration. From these data the approximate binding capacity based on recovery was found to be 1 µg of beads to 100 µg of total protein.

To test binding in the presence of contaminating substances, reduced and alkylated yeast lysates were used. Prior to treatment with SP3, lysates were spiked with the specific contaminating substance (e.g. 10% SDS). Mixtures were treated with SP3 using 50% acetonitrile under acidic conditions with rinses of 70% ethanol (2X) and 100% acetonitrile (1X) as described above. Recovered proteins were digested and the resulting peptides injected to the MS after treatment with peptide SP3 as described

above. Recoveries were compared using MS intensity and chromatogram complexity between samples.

Optimization of SP3 for peptide enrichment

In all peptide experiments, 2 µL of a 10 µg/µL stock of beads was used as discussed in the 'Peptide SP3 Protocol' methods section in the main text. All reactions were performed in PCR tubes (Ratiolab).

To determine the optimum concentration of acetonitrile to promote peptide binding to the beads, aliquots of a peptide mixture (5 µL, ~1 µg) derived from yeast lysate prepared in 1% SDS containing buffer as described above were used. Equivalent bead-peptide mixtures were either acidified using formic acid, or left at neutral pH. To the bead-peptide mixtures, acetonitrile was added from a 100% stock to adjust the final concentration in steps of 5% between 20 – 95% for individual samples. Mixtures were incubated for 8 minutes at room temperature, and then placed on a magnetic rack for a further 2 minutes. Beads were rinsed once with 180 µL of acetonitrile, and peptides eluted off the beads in 10 µL of 2% DMSO with pipette mixing for 5 minutes. Samples were acidified with formic acid prior to MS-analysis. The signal intensity and complexity between runs was compared to determine the optimum percentage of acetonitrile. A final concentration of 95% of acetonitrile in neutral conditions was found to give the highest and most reproducible recovery.

Using this optimized condition, SP3 was compared with conventional methods for peptide clean-up and enrichment. To observe peptide recovery, SP3 was compared with the following treatments: 1. Nanodiamond conditions, 2. SPRI precipitation conditions, 3. StageTip treatment as

described below. In nanodiamond conditions, bead-peptide mixtures were acidified with 5 µL of 1% formic acid. In SPRI conditions, bead-peptide mixtures were mixed with 15 µL of precipitation mix (2.5M NaCl, 20% ammonium sulfate). In both conditions, mixtures were incubated for 8 minutes at room temperature, and then placed on a magnetic rack for a further 2 minutes. Beads were rinsed once with 180 µL of acetonitrile, and peptides eluted off the beads in 10 µL of 2% DMSO with pipette mixing for 5 minutes. Samples were acidified with formic acid prior to MS-analysis. The signal intensity and complexity between runs was compared in the resultant data.

Chemical Labeling of Peptides in SP3

Working solutions of 1% formaldehyde (Thermo Scientific) and 155mM sodium cyanoborohydride (Sigma) and sodium cyanobrodeuteride (Sigma) were prepared in water. Peptide solutions derived from SP3 digests were typically contained in a total volume of 5 µL of digestion buffer, including beads. To each tube, 1 µL of the appropriate 1% formaldehyde solution (light-CH_2O, medium-CD_2O, heavy-$^{13}CD_2O$) was added. In addition, a further 1 µL of sodium cyanoborohydride (light and medium) or sodium cyanobrodeuteride (heavy) was added, and reactions pipette mixed. Labeling reactions were incubated for 1 hour at room temperature. Reactions were quenched through addition of 1 µL of a mixture of 10mM lysine (Sigma) and 50mM ammonium bicarbonate (Sigma). Labeled peptides were treated directly with SP3 peptide clean-up prior to MS analysis.

TMT labeling kits were obtained from Pierce. Each TMT label (0.8mg per vial) was reconstituted in 40 μL of acetonitrile. Peptide solutions derived from SP3 digests were typically contained in a total volume of 5 μL of digestion buffer. Labeling reactions were carried out through addition of 20 μg of TMT label per 10 μg of peptide in two volumetrically equal steps of 1 μL, 30 minutes apart. TMT labeling reactions were carried out on the magnetic rack at all times to avoid potential interactions between peptides and the beads due to the addition of acetonitrile. Reactions were quenched through addition of 1 μL of a mixture of 10mM lysine (Sigma) and 50mM ammonium bicarbonate (Sigma). Labeled peptides were treated directly with SP3 peptide clean-up prior to MS analysis.

Alternative Peptide Clean-up Procedures

When SP3 was not used, peptides were desalted and concentrated using either SepPak or StageTip treatment. For SepPak clean-up, cartridges (50mg C18-t, Waters) were rinsed twice with 1mL of acetonitrile with 0.1% formic acid. Cartridges were then rinsed twice with 1mL of water with 0.1% formic acid prior to sample loading. Loaded samples were rinsed twice with 0.1% formic acid and eluted with 1 mL of 80% acetonitrile containing 0.1% formic acid. StageTips were prepared as previously described(Rappsilber *et al*, 2003). StageTips were rinsed and eluted using the same conditions as with SepPaks. In both cases, eluted samples were concentrated in a SpeedVac centrifuge (Thermo Scientific) and subsequently reconstituted in 0.1% formic acid.

High-pH Reversed Phase Fractionation

High-pH reversed phase analysis was performed either on an Agilent 1200 HPLC system equipped with a variable wavelength detector (254nm) or on StageTips. On the HPLC, fractionation was performed on an XBridge BEH C18 column (1 x 100mm, 3.5 µm, 130Å, Waters). Elution was performed at a flow rate of 0.1mL per minute using a gradient of mobile phase A (20mM ammonium formate, pH 10) and B (acetonitrile), from 1% to 37.5% over 61 minutes. Fractions were collected every 2 minutes across the entire gradient length and concatenated into 10 or 12 final samples as discussed previously(Yang & Shen, 2012). Fractions were dried in a SpeedVac centrifuge and reconstituted in 0.1% formic acid prior to MS analysis. For StageTip fractionation, tips were rinsed 2 times with 100% methanol and 2 times with 20mM ammonium formate (pH 10) prior to sample loading. Fractions were eluted step-wise at acetonitrile concentrations of 11.1%, 14.5%, 17.4%, 20.8%, and 85% in 20 mM ammonium formate (pH 10). Fractions 1 and 5 were combined prior to analysis. Each fraction was dried in a SpeedVac and reconstituted in 0.1% formic acid prior to MS analysis.

SP3 Fractionation

After proteolysis of HeLa lysates, the concentration of acetonitrile in the mixture was adjusted to >95% as in the peptide SP3 protocol. Peptides were selectively eluted from the beads using a first elution at an acetonitrile concentration of 87% (pH ~10 with ammonium formate). The acetonitrile elution was performed by addition of 100 µL of mobile phase to the tube while on the magnetic stand, with gentle pipette mixing over the bead surface for approximately 30 seconds to promote elution. The recovered

eluate was dried in a SpeedVac centrifuge before reconstitution in a mixture of 2% DMSO and 1% formic acid. The final elution step was performed using 10 µL of 2% DMSO with pipette mixing for 30 seconds. The final elution step was acidified with formic acid prior to MS-analysis.

Mass Spectrometry Analysis

For optimization of the SP3 protocol, samples were run on a high-capacity trap (HCT) Ultra Ion Trap MS (Bruker Daltonics). Full scan MS spectra were acquired with a mass range of 350 – 1500 m/z in profile mode. The maximum fill time was set to 200 milliseconds with a target value of 2e5. Fragment-MS spectra were acquired over the mass range of 100 – 2000 m/z with a maximum fill time of 30 milliseconds. All HCT data were processed manually offline using in-house scripts.

For analysis using the qExactive MS (Thermo Scientific) with higher-energy collisional dissociation (HCD) fragmentation, samples were introduced using an UltiMate 3000 LC system (Dionex). Columns used for trapping and separations were packed in-house. Trapping columns were packed in 100 µm internal diameter capillaries to a length of 20 mm with C18 beads (Reprosil-Pur, Dr. Maisch, 5 µm particle size). After trapping, gradient elution of peptides was performed on a C18 (Reprosil-Pur, Dr. Maisch, 3 µm particle size) column packed in-house in 75 µm internal diameter capillaries to a length of 30 cm. Elution was performed with a gradient of mobile phase A (99.9% water and 0.1% formic acid) to 25% B (99.9% acetonitrile and 0.1% formic acid) over 50 minutes, and to 40% B over 15 minutes, for a final length of 90 minutes. Alternatively, the gradient was ramped to 25% B over 100 minutes, and to 40% B over 24 minutes, for a final length of 145

minutes. For TMT labeled samples, the gradient was adjusted to run to a concentration of 27% mobile phase B at the 100-minute step. *Drosophila* embryo single-shots were ramped to 25% B over 200 minutes, and to 40% B over 24 minutes, for a final length of 240 minutes.

Data acquisition on the qExactive MS was carried out using a data-dependent method. The top 12 precursors were selected for tandem-MS/MS (MS2) analysis after HCD fragmentation. Survey scans covering the mass range of 350 – 1500 were acquired at a resolution of 70,000 (at m/z 200), with a maximum fill time of 32 milliseconds, and an automatic gain control (AGC) target value of 1e6. MS2 scans were acquired at a resolution of 17,500 (at m/z 200), with a maximum fill time of 200 milliseconds, and an AGC target value of 5e4. An isolation window of 2.0 m/z with a fixed first mass of 110.0 m/z we applied in all experiments. HCD fragmentation was induced with a normalized collision energy (NCE) of 25 for unlabeled and dimethyl samples and 33 for TMT peptides. The underfill ratio was set at 40% to achieve an intensity threshold of 1e5. Dynamic exclusion was set to exclude the previously selected precursors for a total of 30 or 60 seconds, depending on gradient length. Charge state exclusion was set to ignore unassigned, 1, 5 – 8, and >8 charges. Isotope exclusion was enabled and peptide match was disabled. All data were acquired in profile mode.

Mass Spectrometry Data Analysis

Data-dependent data were analyzed using MaxQuant (ver. 1.4.1.2)(Cox & Mann, 2008). Raw data were searched using an initial tolerance of 20ppm and subsequently re-searched at 6ppm following recalibration. Fragment ion tolerances of 20 ppm and 0.5 Daltons were used

for qExactive and Orbitrap Velos data, respectively. Data were searched using the Andromeda engine built into MaxQuant(Cox *et al*, 2011). Carbamidomethylation of cysteine was specified as a fixed modification, with oxidation of methionine and acetylation of the protein N-terminus as variable. In searches with TMT and dimethyl labeled samples, variable modifications of the N-terminus and lysine were specified for masses of +28.0313 (light dimethyl), +32.0564 (medium dimethyl), and +36.0757 (heavy dimethyl), +224.152478 (TMT-0) and +229.1629 (TMT-6plex). In samples requiring quantification, re-quantify was enabled. Matching between runs was enabled in all searches with specified match and time windows of 2 and 20 minutes, respectively. Identifications were filtered based on a minimum peptide length of 7 amino acids, required false-discovery rate (FDR) of 0.01 at both the peptide-spectral match and protein level based on a target-decoy approach, and a minimum of 1 unique peptide by sequence. The databases searched were complete proteomes including isoforms obtained from UniProt (Human: 88933, *Drosophila*: 41509, Yeast: 6767 total sequences). All databases were appended with a list of common contaminants (cRAP, The GPM).

Bioinformatic and Statistical Analyses

Data sets generated in MaxQuant were exported and analyzed with a combination of scripts built in Python and R designed in-house. Contaminant and decoy proteins were removed from all data sets prior to analysis. Unless stated otherwise, quantification was performed at the peptide level as discussed previously(Karp *et al*, 2010). Briefly, peak areas and annotation information for unique peptides were combined into an expression set object and treated with a generalized-logarithm transformation using the VSN

package (R Bioconductor). The VSN transformation addresses heterogeneity of variance across the dynamic range of peptide abundance. Aggregated protein levels were then calculated using a 20% trimmed mean for all uniquely assigned peptides. Statistical analysis of differential protein expression was performed using Limma(Smyth, 2004) to generate a linear model for estimating fold changes and standard errors prior to empirical Bayes smoothing. Results were adjusted for multiple testing using the Benjamini and Hochberg method. Peptides were required to be quantified as heavy-light pairs in a minimum of 6 (2-4:10-12, 2-4:internal standard hour singles), 1 (2-4:10-12, 2-4:internal standard, 10-12:internal standard, 2-2.5:internal standard, and 3.5-4:internal standard hour pools), and 2 (2-2.5:internal standard and 3.5-4:internal standard hour singles) biological replicates.

Gene ontology analyses were performed as described previously(Cox & Mann, 2012), using a Wilcoxon-Mann-Whitney test to asses differential term-enrichment with the Benjamini-Hochberg method to control for multiple hypothesis testing. The position score for each enriched term was calculated as described previously(Cox & Mann, 2012). Briefly, the position score measures where the center of the distribution of fold change values assigned within a category fall in relation to the total set. Possible values are between -1 and 1 and denote groups of proteins enriched in negative or positive fold change values compared with the total data set. Ontology terms were assigned based on Flybase gene accession numbers using a combination of biomaRt and GO.db packages built into R.

RNA-seq expression values were taken from previously published research(Graveley *et al*, 2011). RPKM values were summarized into expression bins based on the classifications 0-'No/Extremely low', 1-'Very low', 2-'Low', 3-'Moderate', 4-'Moderately high', 5-'High', 6-'Very high', 7-'Extremely high' (Flybase). Only mRNA values from 2-4 and 10-12 hour embryo stages from modENCODE high-throughput RNA-seq experiments were utilized in these analyses. Values were compared using Flybase gene accessions.

Supplementary Protocols

Reagents & Equipment

- Sera-Mag Speed Beads A: (Thermo Scientific; CAT No. 09-981-121, Magnetic Carboxylate Modified).
- Sera-Mag Speed Beads B: (Thermo Scientific; CAT No. 09-981-123, Magnetic Carboxylate Modified).
- Embryo lysis buffer: 5mM EDTA, 5mM EGTA, 10mM NaOH, 1X Protease Inhibitor Cocktail, 10mM HEPES (pH 8.5), 10mM DTT, 1% SDS
- Magnetic stand: needs to be capable of holding PCR tubes. We use racks manufactured in house.

Protocol: Bead Preparation

1. To prepare beads, remove from fridge and keep at room temperature for 10 minutes. Combine 20 µL of Sera-Mag A and 20 µL of Sera-Mag B.
2. Add 160 µL of water.
3. Place PCR tube with beads on a magnetic rack and let beads settle for 2 minutes. Once beads have settled, remove and discard supernatant.
4. Rinse beads with 200 µL of water by pipette mixing (off the magnetic stand). Repeat this rinse two further times.
5. Store beads in 100 µL of water in the fridge. Never freeze the beads.

6. Use 2µL of bead-mix per sample (enough beads for 50 samples); make sure the beads are well resuspended in the solution (vortex).

   Prepared beads can be stored at 4°C indefinitely.

Protocol: Cell Lysis and Protein Preparation for Drosophila Embryos

1. Reconstitute each embryo in 20 µL of embryo lysis buffer and ensure the embryo is contained in liquid at the bottom of the PCR tube.
2. Add 2 µL of the bead stock to each tube.
3. Add 20 µL of TFE to each tube and ensure the embryo is contained in the liquid.
4. Sonicate the tubes for 15 minutes in a Bioruptor cycling in 30-second intervals on the setting ‘high’. The Bioruptor should be operated in the absence of active cooling to facilitate heating of the bath. In addition to aiding in lysis, sonication will shear the chromatin. Degradation of chromatin is essential using either sonication or enzyme treatment (e.g. Benzonase), as it will disrupt the SP3 protocol.
5. Add 0.75 µL of 0.1% formic acid to each tube and pipette mix.
6. Heat each tube for 5 minutes at 95°C, and remove tubes to ice for 30 seconds.
7. Incubate for 30 minutes at 45°C.
8. Add 5 µL of 400 mM iodoacetamide in 50 mM HEPES pH8.5 (36 mg in 500 µL) and incubate for 30 minutes at 24°C.
9. Add 5 µL of 200 mM dithiothreitol to quench the reaction.

The lysate is now ready to be prepared for proteolytic digestion using the SP3 protocol. The lysate can be frozen at this point and stored until use.

Protocol: SP3 Protein Clean-up

The steps described here are based on the use of 10 µL of lysate from the cell preparation protocol that already contains the required 2µL of prepared beads. If you are using a mixture that does not already contain beads, add an appropriate amount (e.g. 20 µg or 2 µL from the 10 µg/µL stock) directly to the mixture.

1. Add 5 µL of 1% formic acid to the protein lysate and check that the mixture is acidic.
2. Immediately add acetonitrile to obtain a final percentage of 50% (15 µL of a 100% stock). **Alternate Protocol**: a pre-made mix of formic acid and acetonitrile can be used and added as a single step.
3. Incubate for 8 minutes at room temperature off the rack.
4. Place on magnetic rack and incubate for further 2 minutes at room temperature.
5. Remove and discard supernatant.
6. Add 200 µL of 70% ethanol and incubate for 30 seconds on the magnetic stand. Remove and discard supernatant.
7. Add 200 µL of 70% ethanol and incubate for 30 seconds on the magnetic stand. Remove and discard supernatant.

8. Add 180 µL of acetonitrile and incubate for 15 seconds on the magnetic stand. Remove and discard supernatant and air-dry the beads for 30 seconds.
9. Reconstitute beads in water (or desired buffer of choice). For digestion, reconstitute beads in 5 µL of digestion solution (e.g. 50 mM HEPES pH 8.0 + Xµg of trypsin/lysC mix (1:25 enzyme to substrate ratio)). It is not necessary to completely homogenize the beads, as they will be stuck to each other and excessive handling will generate bubbles. The cluster will break down as proteins are digested.
10. Incubate for 14 hours at 37°C.

Digested peptides can be recovered from the beads by placing the tube on a magnetic rack and removing the supernatant containing them. The peptide mixture can be used directly in downstream fractionation, labeling, or conventional clean-up workflows. The beads can be sonicated to improve recovery of the peptides.

Protocol: SP3 Peptide Recovery and Preparation

This protocol is based on the use of 5 µL of protein digest that already contains beads. If you are using a digest that does not already contain beads, add an appropriate amount (e.g. 20 µg or 2 µL from the 10 µg/µL stock) directly to the peptide mixture.

1. Resuspend beads after digestion by pipetting.
2. Add acetonitrile to each sample to obtain a final percentage of 95% or higher and pipette mix. Incubate for 8 minutes at room temperature.

3. Place on magnetic rack for a further 2 minutes.
4. Remove and discard supernatant.
5. Add 180µL of acetonitrile and incubate for 15 seconds on the magnetic stand. Remove and discard supernatant.
6. Reconstitute beads in desired volume of 2% DMSO (in water, not acidic) (ex. 9 µL). **Optional:** sonicate the reconstituted beads for 1 minute to improve recovery.
7. Give the tube a quick spin (~2 seconds) in a bench-top PCR-tube centrifuge to aid the liquid removal from the tube walls.
8. Place tubes on a magnetic rack and recover supernatant making sure to not recover any beads. **Optional:** To eliminate any potential bead carryover, it is recommended that the removed liquid be transferred to a fresh PCR tube, placed on the magnetic rack, and recovered a second time with a fresh pipette tip.
9. Place supernatant in a deactivated glass vial containing 1% formic acid (ex. 1 µL).
10. Mix by pipetting and centrifuge for 30 seconds at 5,000 g.

Recovered samples can be directly injected to mass spectrometer hardware without prior treatment. Samples can be stored at -20°C prior to, and after acidification.

Supplementary Figures

(a)

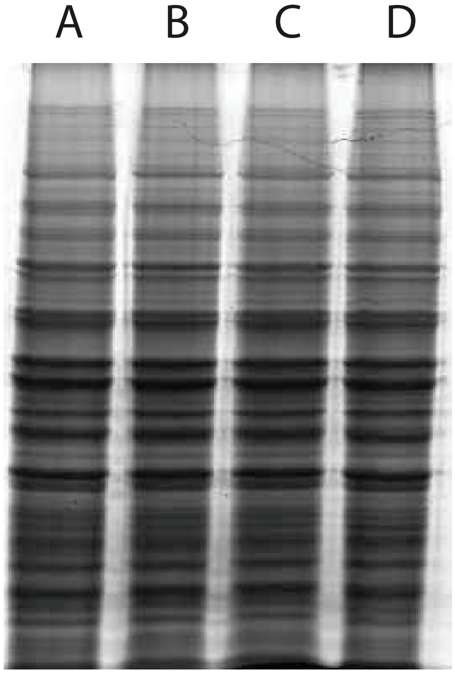

(b)

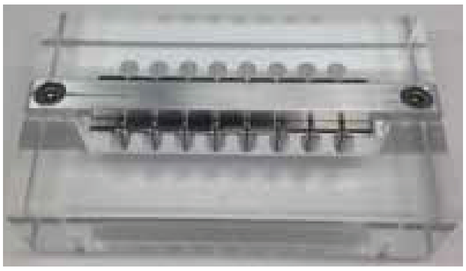

16-position Magnetic Rack
(PCR Tubes)

(c)

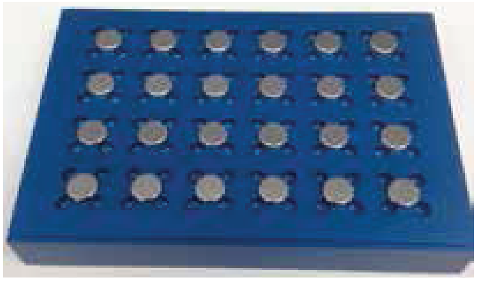

96-position Magnetic Rack
(PCR Plates)

Supplementary Figure S1 – SP3 can be performed in a wide variety of conditions with beads from separate manufacturers. (a) Coomassie stained SDS-PAGE gel analysis of a yeast cell lysate (~40 µg of protein) that remained untreated (A), or was prepared with carboxylate beads from two separate manufacturers: Ampure XP from Beckman Coulter (B) and PCR Clean from CleanNA (C and D). The CleanNA beads are used in two separate conditions, with poly-ehtylene glycol (C) as a precipitation agent or ammonium sulfate (D). (b-c) Magnetic racks used in all SP3 workflows. Racks are manufactured in-house and are compatible with 96-well format robotics automation.

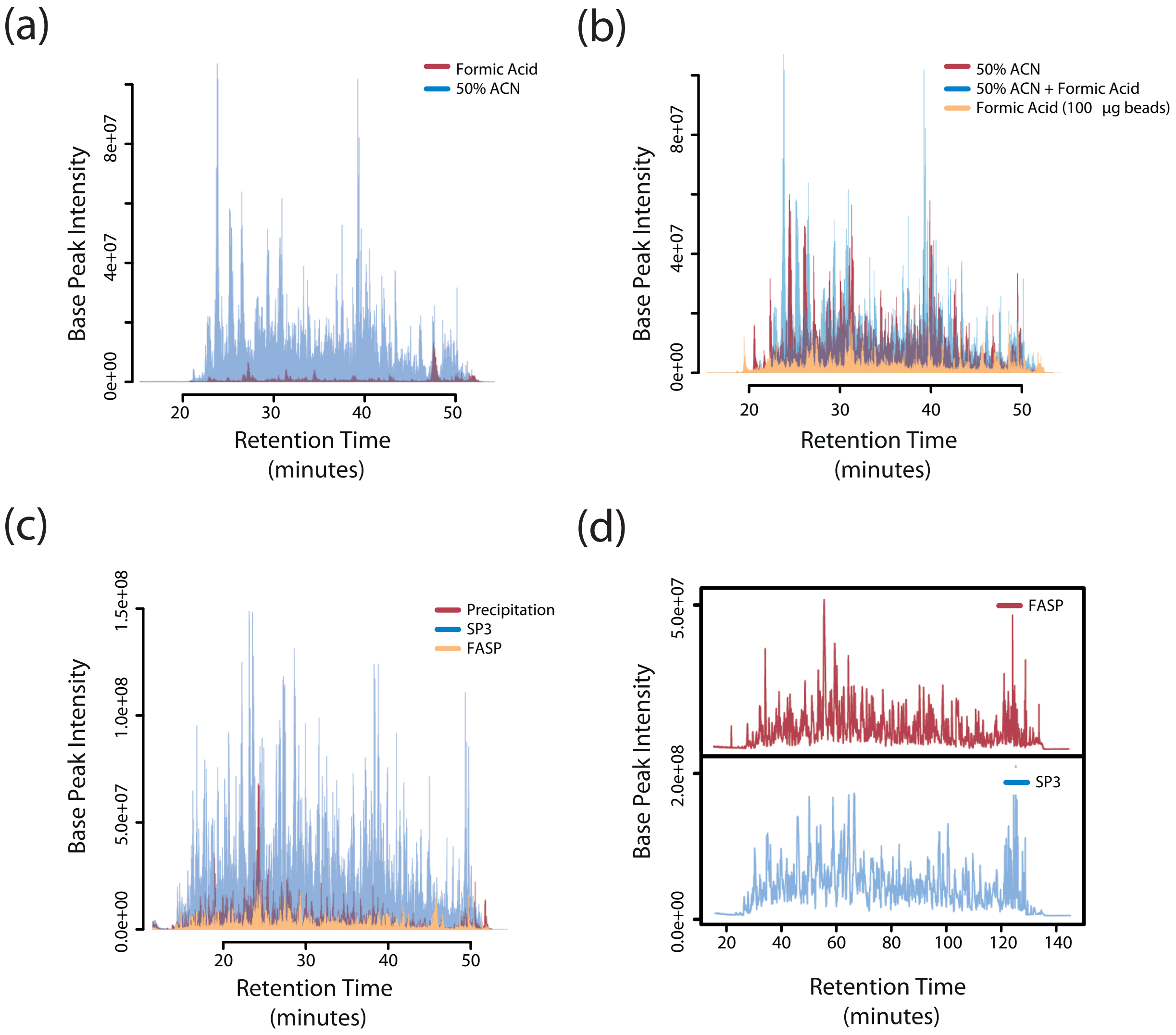

Supplementary Figure S2 – Proteins can be efficiency captured and rinsed using carboxylate modified paramagnetic beads in the SP3 protocol. Equivalent aliquots of a yeast whole-cell lysate (~10 µg of total protein) in SDS-containing buffer were treated with acidic (nanodiamond), acetonitrile in acidic conditions (SP3), and salt (SPRI) conditions. Recovered proteins were digested and the resulting peptides run on an HCT-ion trap MS. (a) SP3 is superior to nanodiamond conditions for protein enrichment. MS base peak chromatograms of a digested yeast lysates. Protein-bead mixtures were treated with nanodiamond (acidic) or SP3 (acidic with 50% acetonitrile) conditions. Bound proteins were equivalently rinsed, digested, recovered and directly injected to the MS. (b) MS base peak chromatograms of yeast lysates treated with SP3 with or without formic acid. Alternatively, mixtures were acidified with formic acid in the presence of a high concentration of beads (100 µg) in the absence of acetonitrile. (c) SP3 is superior to FASP and SPRI conditions for protein enrichment. MS base peak chromatograms of yeast lysates treated with high salt (SPRI), acetonitrile with formic acid (SP3), or with FASP. (d) Extended chromatographic runs of yeast lysates treated with the optimized SP3 protein protocol (50% ACN with formic acid) and FASP.

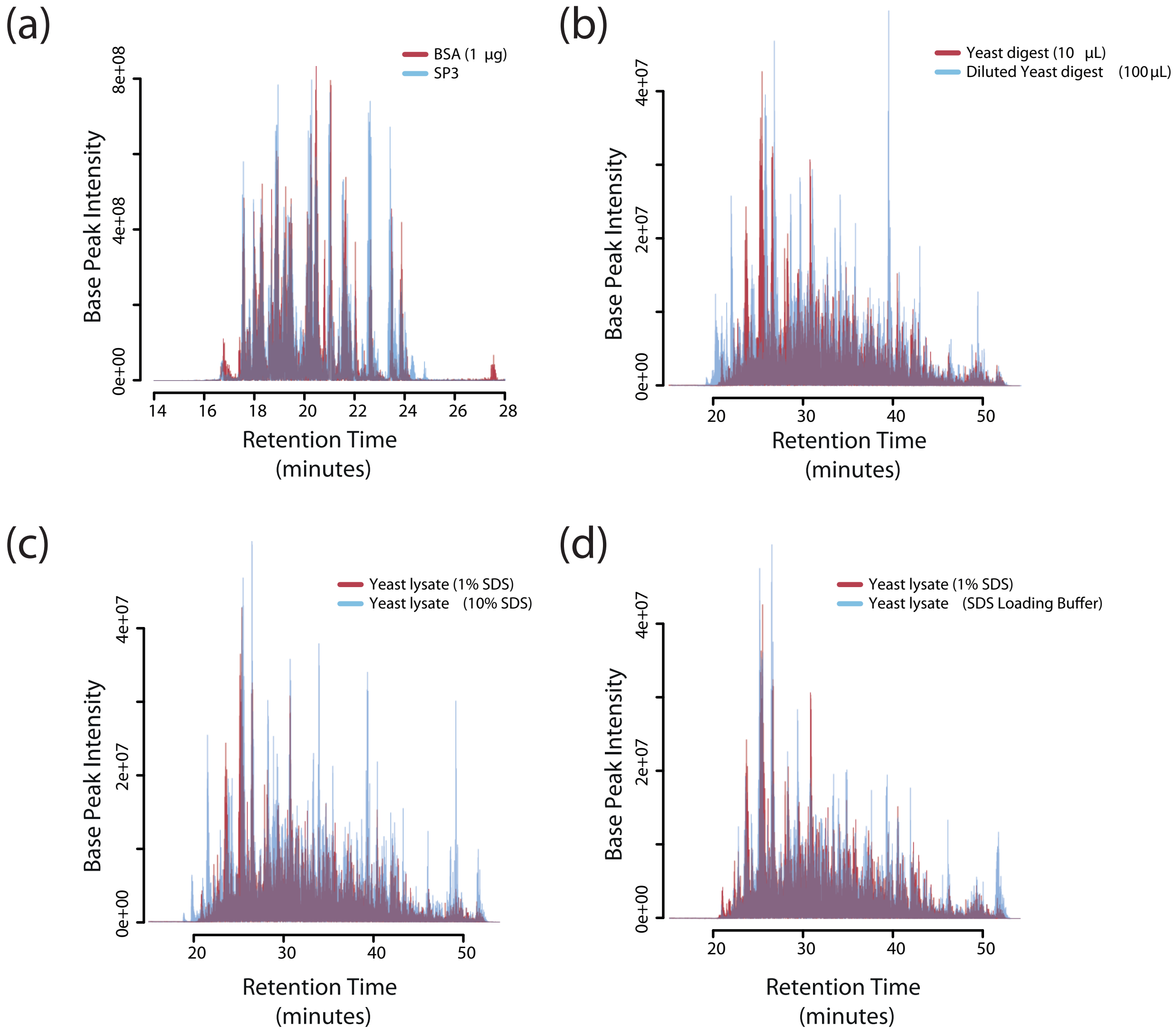

Supplementary Figure S3 – Proteins can be efficiency captured and rinsed with SP3 in diverse conditions. Specified amounts of reduced and alkylated BSA in a 1% SDS buffer were used to determine approximate binding ranges. (a) *Nanoparticles have a capacity of ~100 µg of protein per 1 µg of SP3 beads.* BSA (100 µg) was combined with beads (1 µg) and treated with SP3. Recovered protein was digested, and resulting peptides diluted to inject to the MS. Capacity was measured based on recovery compared to a standard of 1 µg of BSA. (b) *Proteins can be efficiently enriched from dilute or detergent containing solutions.* Equivalent aliquots of a yeast cell lysate were used to determine recovery in dilute solutions, and in the presence of detergents. Overlaid base peak chromatograms depicting recovery of 10 µg a protein mixture treated with SP3 directly, or after dilution to a total volume of 100 µL. An equivalent volume of the recovered peptides was injected to the MS. (c) Overlaid base peak chromatograms depicting recovery of 10 µg of a yeast lysate containing 1% or 10% SDS (v/v) with SP3. (d) Overlaid base peak chromatograms depicting recovery of 10 µg of a yeast lysate containing 1% SDS (v/v) or 1X Laemmli buffer were treated with SP3. Mixtures were equivalently rinsed, digested, and injected to the MS.

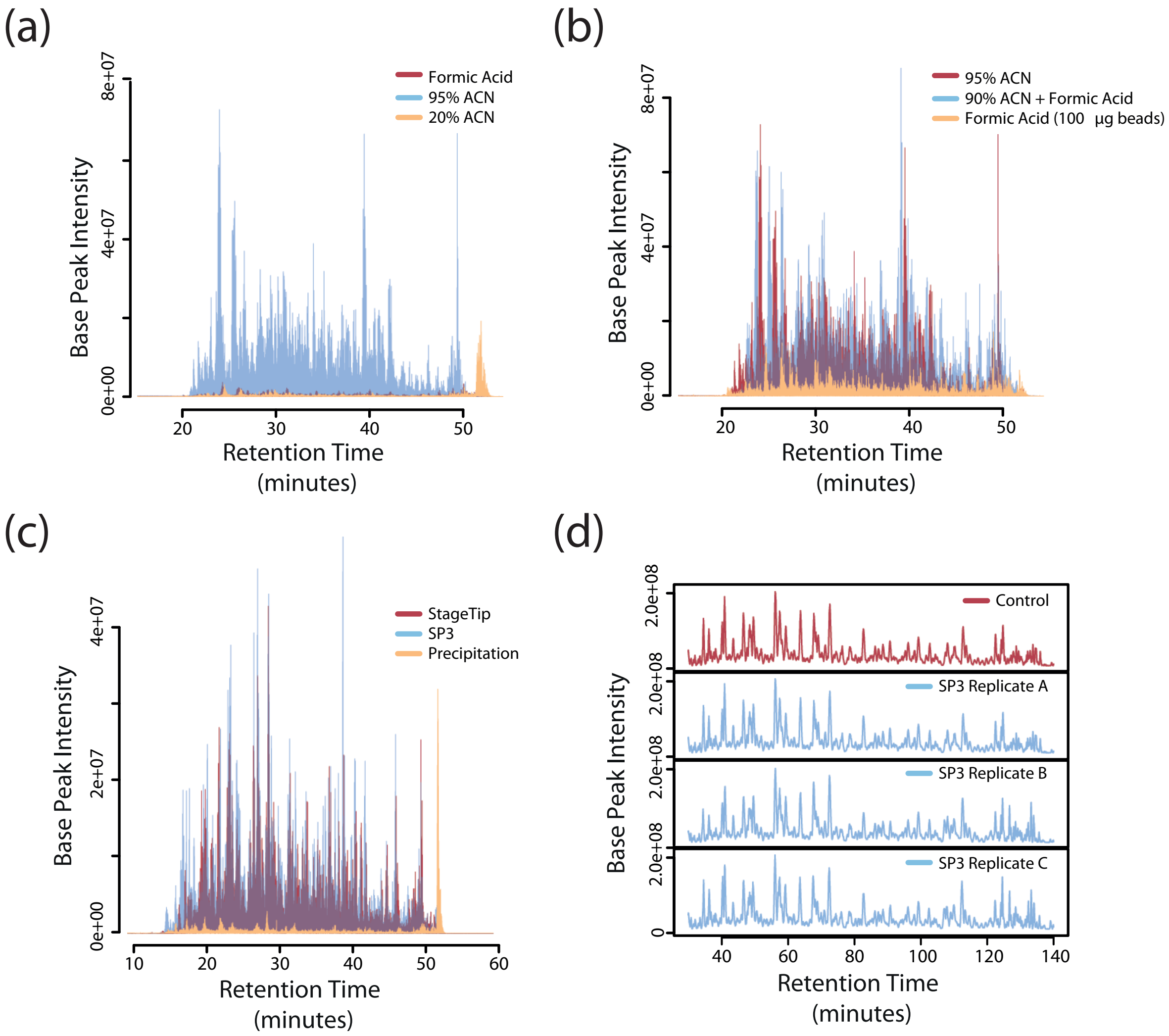

Supplementary Figure S4 – Peptides can be efficiency captured and rinsed with SP3. Equivalent aliquots of a yeast whole-cell lysate digested with trypsin and rLysC were treated with acidic (nanodiamond), acetonitrile (SP3), and salt (SPRI) conditions. (a) *SP3 is superior to nanodiamond conditions for peptide enrichment.* MS base peak chromatograms of recovered peptide mixtures after acidification with formic acid or addition of acetonitrile to a final concentration of 20% or 95% from a 100% stock. (b) MS base peak chromatograms of recovered peptide mixtures after volumetric adjustment to a final concentration of 95% acetonitrile, 90% acetonitrile with formic acid, or treatment with formic acid in the presence of a high amount of beads (nanodiamond). (c) *SP3 is superior to SPRI conditions for peptide enrichment.* MS base peak chromatograms depicting recovery of peptide mixtures treated with high salt (SPRI), 95% acetonitrile (SP3), or StageTips. d) *SP3 is unbiased and reproducible for peptide enrichment.* Chromatrograms depicting recovery from replicate peptide mixtures treated with the optimized SP3 protocol (95% acetonitrile) or StageTips (Control).

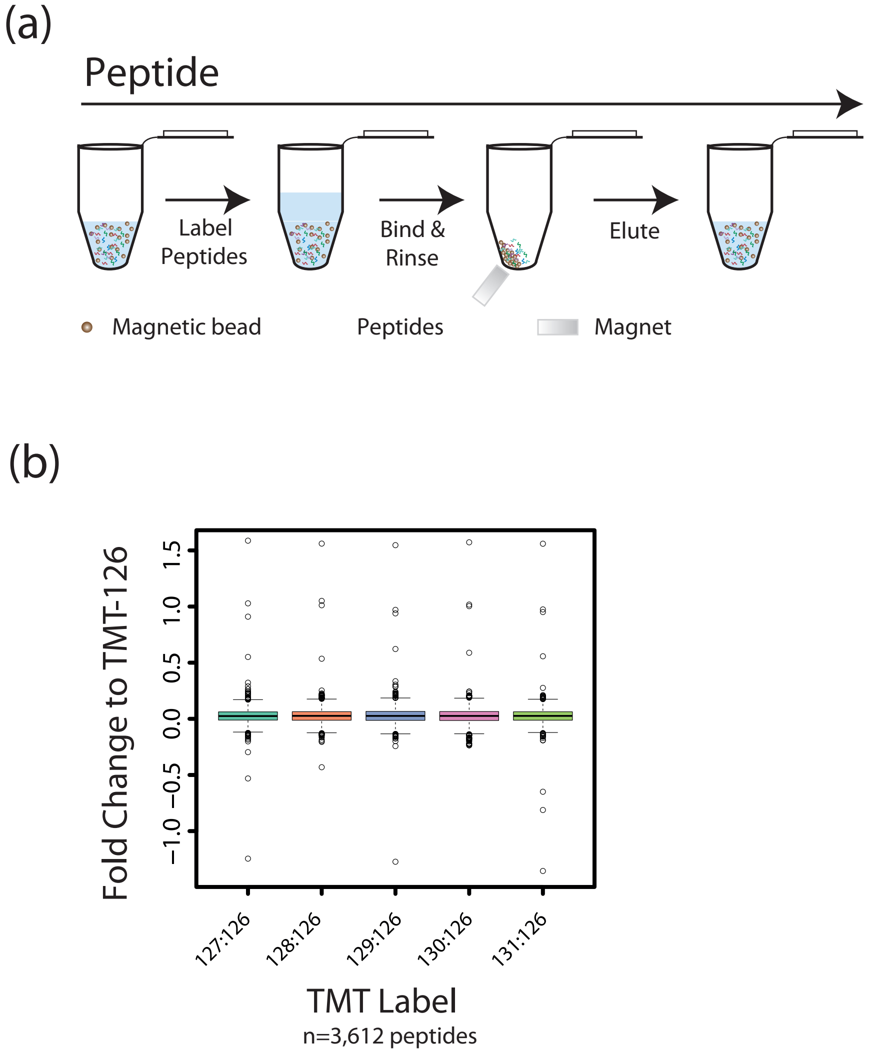

Supplementary Figure S5 – SP3 is compatible with chemical labeling of peptides using dimethyl and TMT approaches. Equivalent digests derived from a yeast-whole cell lysate were treated with dimethyl or TMT labeling protocols in triplicate. (a) Chemical labeling is applied directly after SP3 protein clean-up in the same tube prior to elution and MS analysis. SP3 peptide clean-up is performed with no modification. (b) Reproducibility of labeling as measured by deviance from an expected fold change of 0 for peptides identified in a minimum of 3 channels (n=3,612) in a TMT 6-plex experiment across replicates. Ratios represent VSN normalized peptide-level calculations. Whiskers indicate 1.5x the interquartile range, plus or minus the values for the third or first quartiles, respectively

(a)

	FASP		SP3	
	Replicate A	Replicate B	Replicate A	Replicate B
# of proteins	3998	3995	3928	3934
# of peptides	39684	39054	35973	35813
Median # unique	7	7	7	7
Mean sequence coverage	26.4	25.8	24.6	24.7

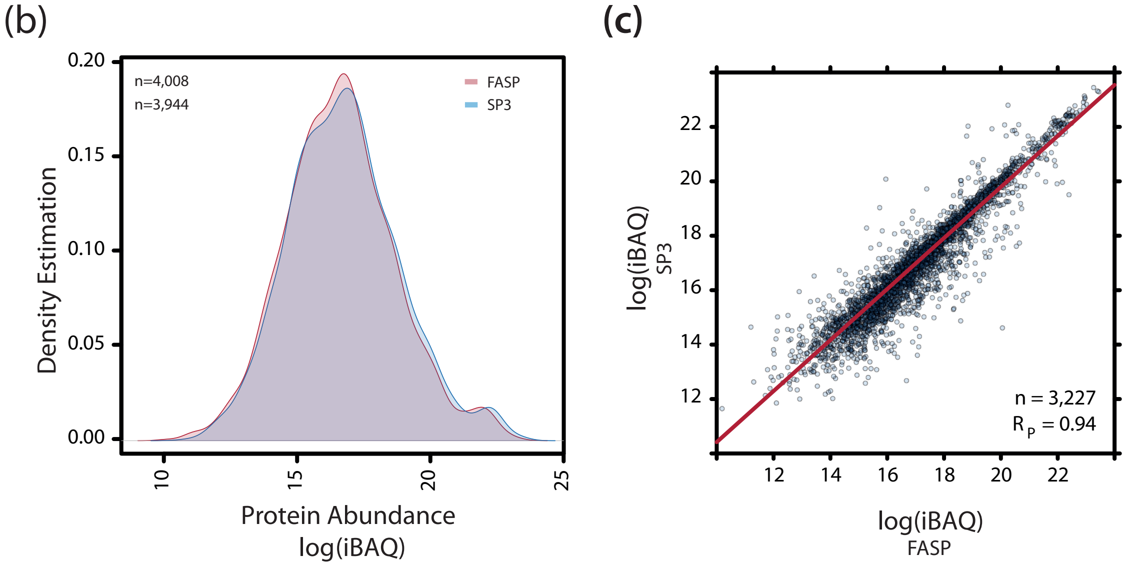

Supplementary Figure S6 – SP3 produces comparable results compared with FASP for in-depth proteome analysis. Duplicate aliquots of a yeast whole-cell lysate (~10 µg) were prepared with SP3 or FASP prior to digestion with trypsin and rLysC. Peptides were fractionated with high-pH reversed phase chromatography and analyzed with a qExactive MS. (a) Protein and peptide identification metrics from the FASP and SP3 data. (b) *There is no discernable difference in the abundance profile based on iBAQ values of proteins identified in FASP and SP3 data.* Distribution of abundance as log (iBAQ) values for all proteins identified between FASP and SP3 samples. (c) Scatter plot of log(iBAQ) values for proteins identified between FASP and SP3 samples. Correlation was determined using the Pearson method. Red line indicates a linear fit to the data.

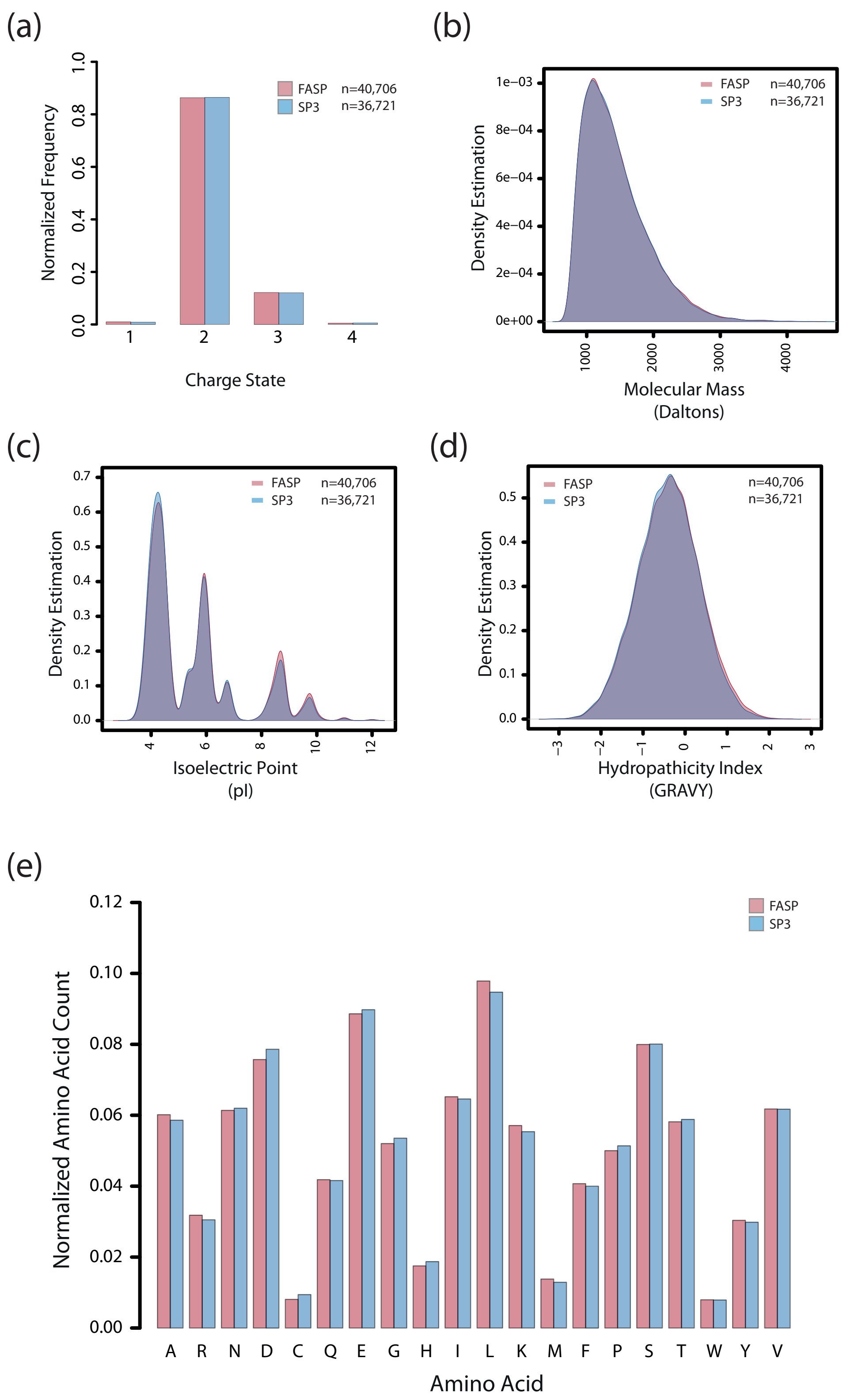

Supplementary Figure S7 – There is no observable bias in the peptides identified with SP3 when compared with those from the FASP approach. Duplicate aliquots of a yeast whole-cell lysate were prepared with SP3 or FASP prior to digestion with trypsin and rLysC. Peptides were fractionated with high-pH reversed phase chromatography and analyzed by MS. Plots represent histograms and kernel density estimations of charge state (a), molecular mass (b), isoelectric point (c), and GRAVY index (d) of identified unique peptides from each method. (e) Barplot of the counts for each residue identified in FASP and SP3 samples illustrating the lack of bias against any specific amino acid. Peptide properties were determined using the ProtParam tool from ExPASy called from a Python script developed in-house.

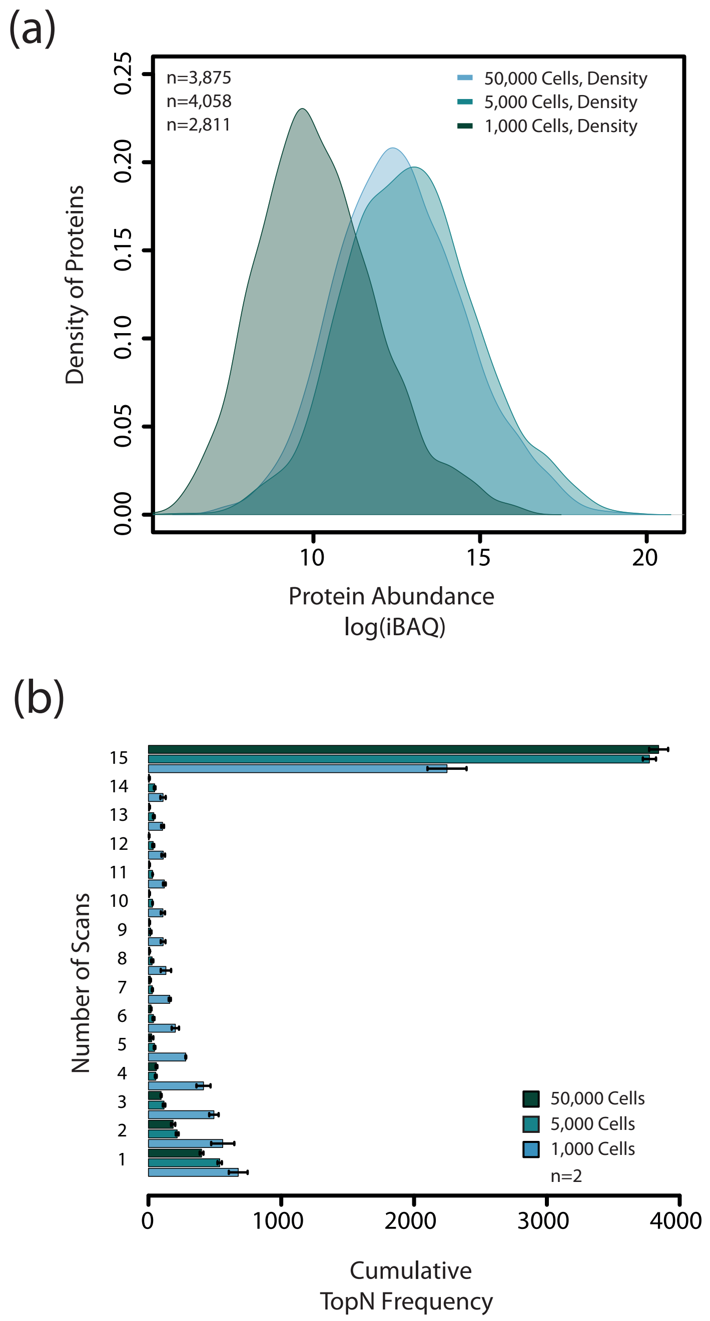

Supplementary Figure S8 – HeLa single shot samples from >5,000 cells display similar proteome complexity and abundance profiles. Replicates of specific starting numbers of HeLa cells were analyzed as single-shot injections in a 4-hour gradient on an Orbitrap Velos MS. (a) Kernel density estimations of fractional protein abundance densities determined using iBAQ values from single-shot runs. Curves depict the higher recovery of total protein in the 50,000 and 5,000 cell samples in relation to the 1,000. (b) Sampling rate as determined by the frequency of TopN scans. A value of 15 indicates the instrument selected the maximum number of precursors during the specified scan event. Data are presented as mean values of biological duplicates (n=2) with standard deviation displayed as error bars.

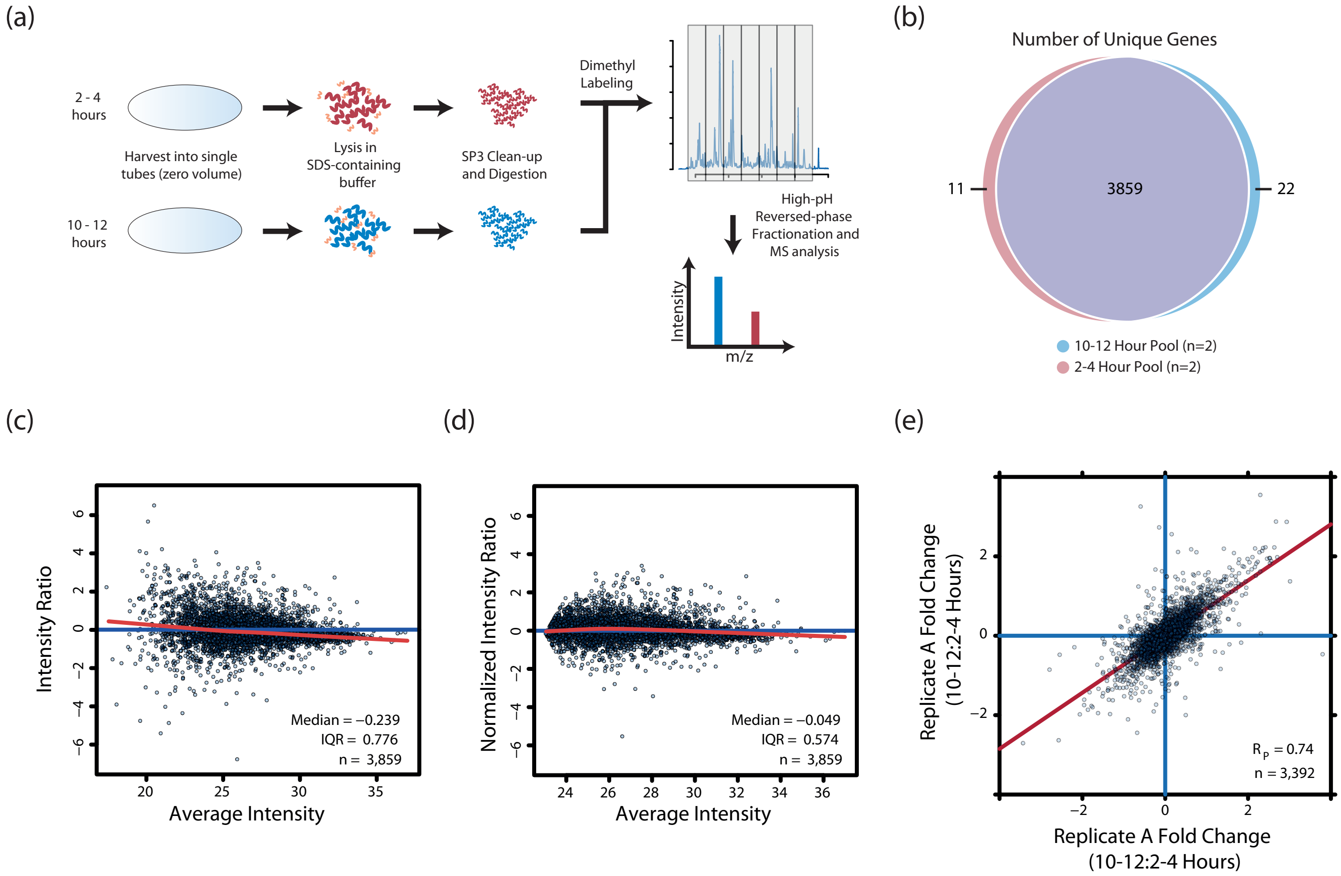

Supplementary Figure S9 – Significant depth and reproducible quantification of the proteome from low numbers of *Drosophila* embryos can be obtained using SP3. (a) Pools of 20 *Drosophila* embryos derived from 2-4 and 10-12 hours harvests (AEL) were prepared with SP3 prior to fractionation with high-pH reversed phase chromatography and analysis using dimethyl tags. (b) Venn diagram depicting the number of identified gene products between the two-pooled samples based on Flybase gene accessions. Data are from combined biological duplicates for each harvest window. *Reproducible quantification from limited samples can be obtained using SP3.* Intensity ratio (M) and average intensity (A) plots of non-transformed (c) and transformed (d) data from embryo pools. N-values represent the number of uniquely identified proteins quantified in the two sample conditions. Data are from combined biological duplicates from each harvest window (n=2). Blue lines indicate a fold-change of zero, and red a locally weighted regression. (e) Scatter plot depicting the reproducibility of quantification between for those proteins identified in both replicates. Correlation values were determined using the Pearson method. Blue lines indicate zero fold change, and red a linear fit to the data.

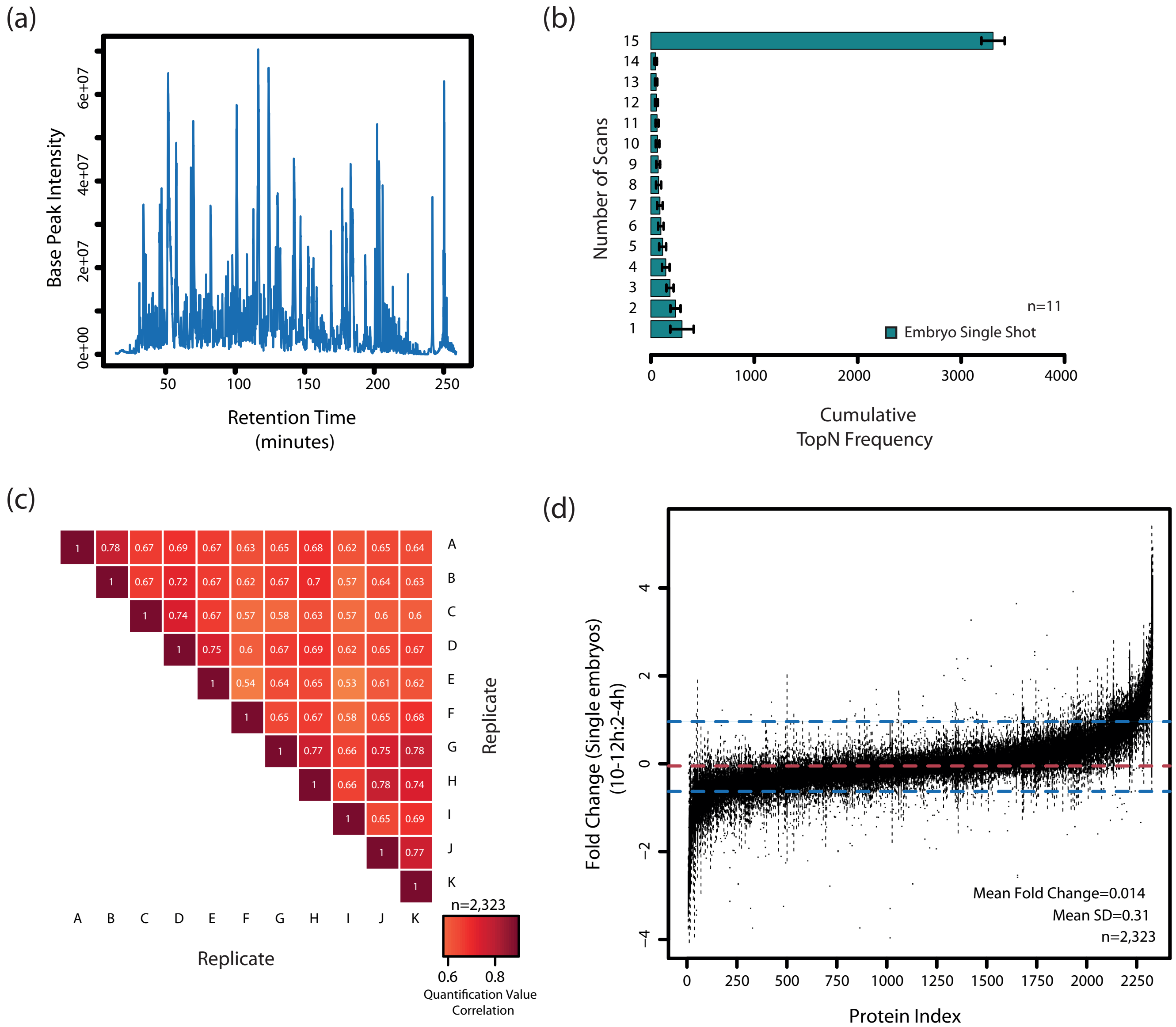

Supplementary Figure S10 – MS analysis of single embryos reveals high complexity of the contained proteome and the reproducible quantification in the obtained data. Harvested and SP3 treated dimethyl labeled embryos were injected as single-shot runs on an Orbitrap Velos MS system. (a) Base peak chromatogram depicting the complexity derived from the single embryo samples. The data illustrate that a significant amount of material can be isolated from a single embryo using SP3. (b) Sampling rate as determined by the frequency of TopN scans. A value of 15 indicates the instrument selected the maximum number of precursors during the specified scan event. Data are presented as mean values of biological replicates (n=11) with standard deviation displayed as error bars. (c) Heat map depicting the correlation for proteins identified and quantified across all biological replicates (n=11). Values in each box depict the correlation coefficient determined using the Pearson method. (d) Boxplot of fold change values for proteins identified and quantified across all biological replicates. Whiskers indicate 1.5x the interquartile range, plus or minus the values for the third or first quartiles, respectively. Dashed red and blue lines indicate the median and a range spanning 5% - 95% of fold-change values.

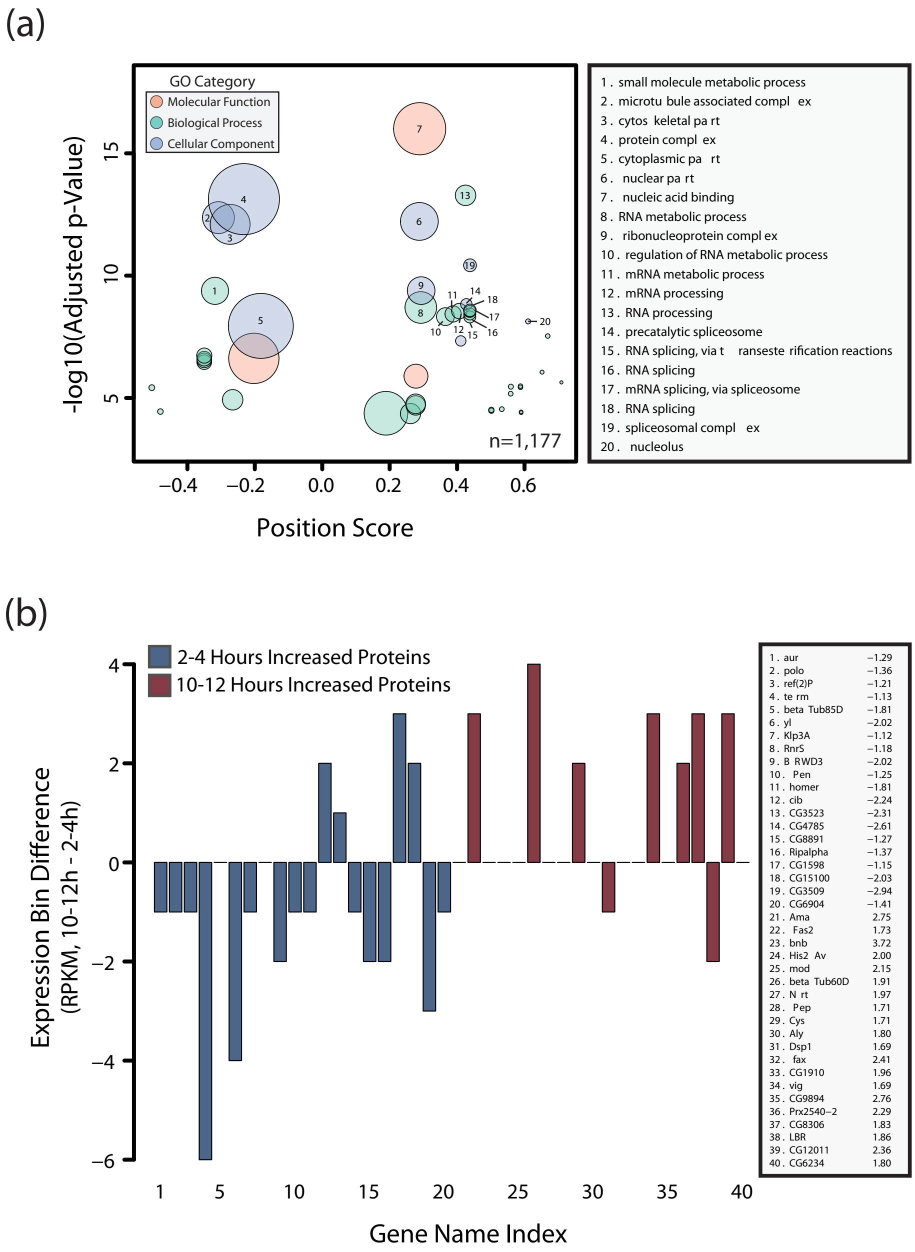

Supplementary Figure S11 – Single embryo analyses reveal diverse changes in the developmental proteome that correspond with changes in gene expression. (a) Gene ontology enrichment analysis for proteins quantified in single embryo samples. Proteins with fold change values between -0.25 and 0.25 were excluded from the analysis (n=1,177 remaining). On the plot, color indicates the gene ontology classification and spot size the number of genes assigned to the category. Gene ontology term assignments were made based on Flybase gene accessions using the GO.db and biomaRt packages in R. P-values were determined using a Wilcoxon-Mann-Whitney test and adjusted for multiple testing using the Benjamini-Hochberg method. Position scores measure the center of the distribution of fold change values assigned within a category in relation to the total set. Values between -1 and 1 denote groups of proteins with positive or negative fold change values in relation to the total data set. The table on the right displays numbered annotations for the top 20 assigned gene ontology classifications based on adjusted p-value in the plot. (b) Bar chart depicting changes in gene expression based on binned RPKM classifications from Flybase. Bin values are classified as 'No/Extremely low' (0) to 'Extremely High' (7). The top 20 increased 2-4 and 10-12 hours genes as determined in the single embryo proteome analyses are displayed. Values are plotted as the difference in bin value between pools (10-12 – 2-4 hours). Colors indicate the developmental stage for each gene expression value. Legend depicts gene names for the corresponding index values. Protein fold change values (10-12:2-4 hours) are displayed beside each gene.

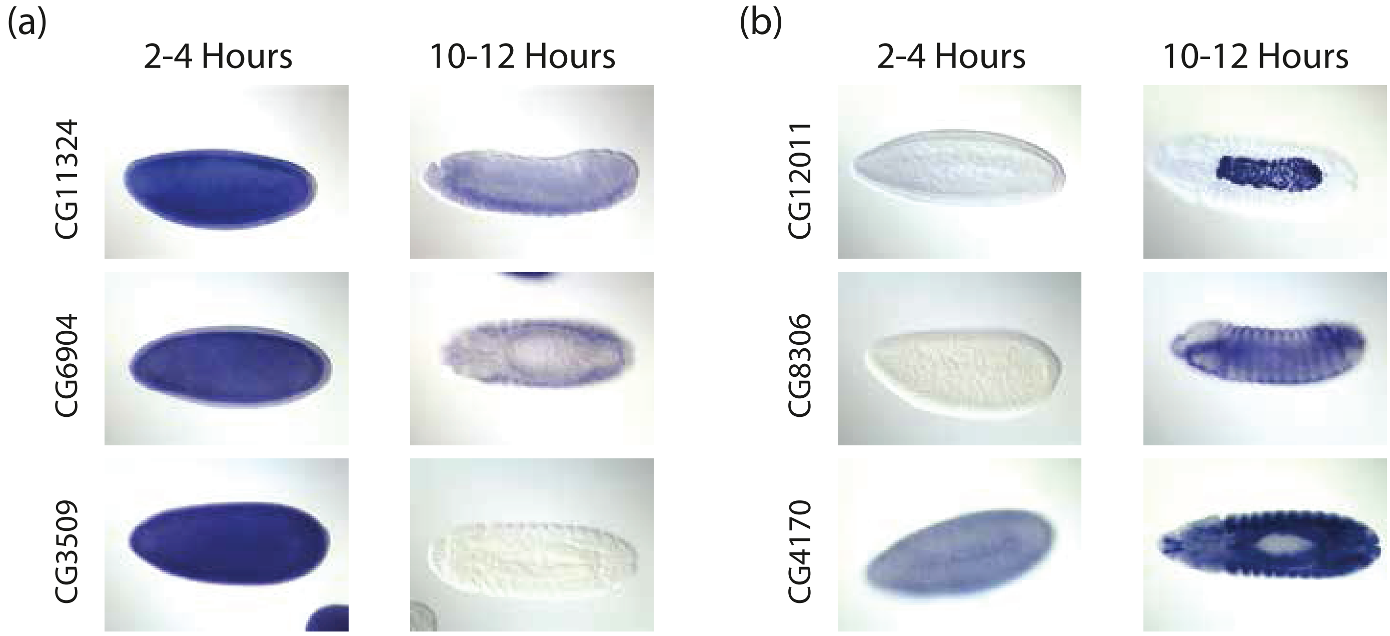

Supplementary Figure S12 – Observed protein expression patterns correspond to changes in RNA abundance. RNA in situ hybridization images displaying expression patterns for selected genes observed to have differential abundance between 2-4 and 10-12 hours stages. Genes enriched in 2-4 (a) or 10-12 (b) hours are displayed. Images are reproduced from the Berkeley Drosophila Genome Project (http://insitu.fruitfly.org/cgi-bin/ex/insitu.pl)(Hammonds et al., 2013; Tomancak et al., 2002, 2007).

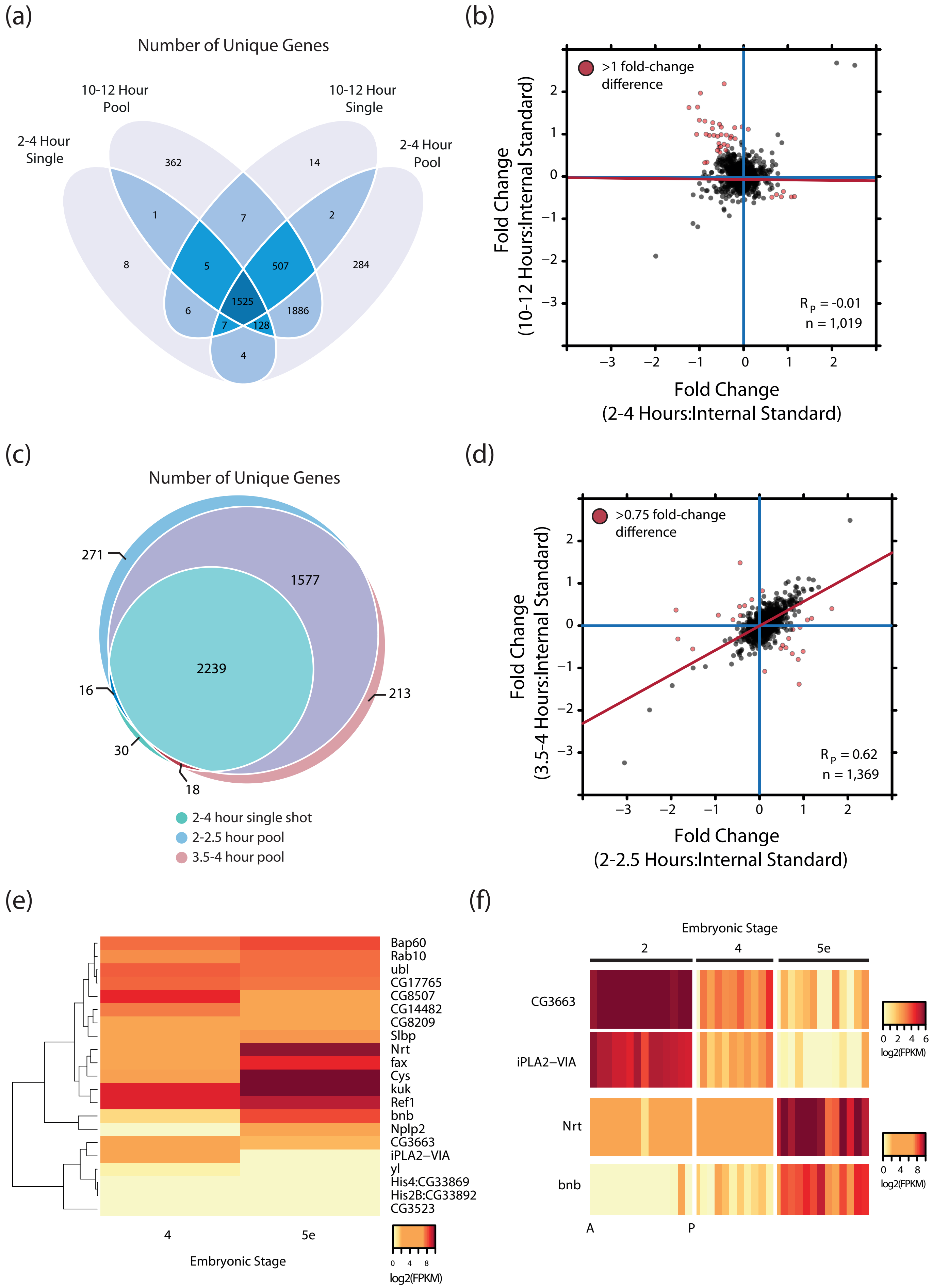
(a)
Number of Unique Genes
10-12 Hour Pool
10-12 Hour Single
2-4 Hour Single
2-4 Hour Pool
362
14
1
7
2
8
5
507
284
1525
6
1886
7
128
4
(b)
>1 fold-change difference
Fold Change (10-12 Hours:Internal Standard)
Fold Change (2-4 Hours:Internal Standard)
R P = -0.01
n = 1,019
(c)
Number of Unique Genes
271
1577
2239
16
213
30
18
2-4 hour single shot
2-2.5 hour pool
3.5-4 hour pool
(d)
>0.75 fold-change difference
Fold Change (3.5-4 Hours:Internal Standard)
Fold Change (2-2.5 Hours:Internal Standard)
R P = 0.62
n = 1,369
(e)
Bap60
Rab10
ubl
CG17765
CG8507
CG14482
CG8209
Slbp
Nrt
fax
Cys
kuk
Ref1
bnb
Nplp2
CG3663
iPLA2–VIA
yl
His4:CG33869
His2B:CG33892
CG3523
4
5e
Embryonic Stage
log2(FPKM)
(f)
Embryonic Stage
2
4
5e
CG3663
iPLA2–VIA
Nrt
bnb
A
P
log2(FPKM)
log2(FPKM)

Supplementary Figure S13 – SP3 provides sufficient depth of coverage and quantitative accuracy to facilitate expression mapping of single embryos. (a) Venn diagram depicting the overlap in the unique gene products identified between the 'low resolution' single embryo and pooled samples. Gene accessions were determined using Flybase. (b) Fold change values between 2-4 and 10-12 hours pooled embryo samples relative to the internal standard. Colored values depict those that have a difference in fold-change >1 between the data sets. Only proteins identified in both pooled data sets as well as in all single embryos are retained (n=1,019). Data are from combined biological duplicates. Blue lines indicate zero fold change, and red a linear fit to the data. (c) Venn diagram depicting the overlap in unique gene products identified between the 'high resolution' single embryo and pooled data sets. (d) Fold change values between 2-5 and 3.5-4 hours pooled embryo samples relative to the internal standard. Colored values depict those that have a difference in fold-change >0.75 between the data sets. Only proteins identified in both pooled data sets as well as in all single embryos are retained (n=1,369). Data are from combined biological duplicates. Blue lines indicate zero fold change, and red a linear fit to the data. Correlation coefficients in (b) and (d) were determined using the Pearson method. (e) Heat map of the 25 candidate proteins chosen for differential expression mapping between the 2 and 4-hour edges of the collection window. Expression values are presented as the logarithm base 2 of the Fragments per Kilobase of Exon per Million Fragments Mapped (FPKM) as reported previously(Combs & Eisen, 2013). The median value for the combined slices was used in all instances. (f) Expression maps for embryo sections as reported previously(Combs & Eisen, 2013). Values are log2(FPKM) and are represented in the anterior (A) to posterior (P) direction for the selected genes.

Supplemental References

Combs P a & Eisen MB (2013) Sequencing mRNA from cryo-sliced Drosophila embryos to determine genome-wide spatial patterns of gene expression. *PLoS One* **8:** e71820

Cox J & Mann M (2008) MaxQuant enables high peptide identification rates, individualized p.p.b.-range mass accuracies and proteome-wide protein quantification. *Nat. Biotechnol.* **26:** 1367–72

Cox J & Mann M (2012) 1D and 2D annotation enrichment: a statistical method integrating quantitative proteomics with complementary high-throughput data. *BMC Bioinformatics* **13 Suppl 1:** S12

Cox J, Neuhauser N, Michalski A, Scheltema R a, Olsen J V & Mann M (2011) Andromeda: a peptide search engine integrated into the MaxQuant environment. *J. Proteome Res.* **10:** 1794–805

Graveley BR, Brooks AN, Carlson JW, Duff MO, Landolin JM, Yang L, Artieri CG, van Baren MJ, Boley N, Booth BW, Brown JB, Cherbas L, Davis C a, Dobin A, Li R, Lin W, Malone JH, Mattiuzzo NR, Miller D, Sturgill D, et al (2011) The developmental transcriptome of Drosophila melanogaster. *Nature* **471:** 473–9

Gruhler A, Olsen J V, Mohammed S, Mortensen P, Faergeman NJ, Mann M & Jensen ON (2005) Quantitative phosphoproteomics applied to the yeast pheromone signaling pathway. *Mol. Cell. Proteomics* **4:** 310–27

Hammonds AS, Bristow C a, Fisher WW, Weiszmann R, Wu S, Hartenstein V, Kellis M, Yu B, Frise E & Celniker SE (2013) Spatial expression of transcription factors in Drosophila embryonic organ development. *Genome Biol.* **14:** R140

Karp N a, Huber W, Sadowski PG, Charles PD, Hester S V & Lilley KS (2010) Addressing accuracy and precision issues in iTRAQ quantitation. *Mol. Cell. Proteomics* **9:** 1885–97

Rappsilber J, Ishihama Y & Mann M (2003) Stop and go extraction tips for matrix-assisted laser desorption/ionization, nanoelectrospray, and LC/MS sample pretreatment in proteomics. *Anal. Chem.* **75:** 663–70

Smyth GK (2004) Linear models and empirical bayes methods for assessing differential expression in microarray experiments. *Stat. Appl. Genet. Mol. Biol.* **3:** Article3

Tomancak P, Beaton A, Weiszmann R, Kwan E, Shu S, Lewis SE, Richards S, Ashburner M, Hartenstein V, Celniker SE & Rubin GM (2002) Systematic determination of patterns of gene expression during Drosophila embryogenesis. *Genome Biol.* **3:**

Tomancak P, Berman BP, Beaton A, Weiszmann R, Kwan E, Hartenstein V, Celniker SE & Rubin GM (2007) Global analysis of patterns of gene expression during Drosophila embryogenesis. *Genome Biol.* **8:** R145

Wisniewski J, Zougman A, Nagaraj N & Mann M (2009) Universal sample preparation method for proteome analysis. *Nat. Methods* **6:** 3–7

Yang F & Shen Y (2012) High-pH reversed-phase chromatography with fraction concatenation for 2D proteomic analysis. *E Rev. Proteomics* **9:** 129–134
